# SciKit-SurgeryGlenoid, an Open Source Toolkit for Glenoid Version Measurement

**DOI:** 10.1117/12.2608597

**Published:** 2022-04-04

**Authors:** Asta Olafsdottir, Addie Majed, David Butt, Mark Falworth, Matthew J. Clarkson, Stephen Thompson

**Affiliations:** 1 Wellcome/EPSRC Centre for Interventional and Surgical Science, University College London, United Kingdom; 2 The Royal National Orthopaedic Hospital NHS Trust

## Abstract

Correct understanding of the geometry of the glenoid (the socket of the shoulder joint) is key to successful planning of shoulder replacement surgery. This surgery typically involves placing an implant in the shoulder joint to restore joint function. The most relevant geometry is the glenoid version, which is the angular orientation of the glenoid surface relative to the long axis of the scapula in the axial plane. However, measuring the glenoid version is not straightforward and there are multiple measurement methods in the literature and used in commercial planning software.

In this paper we introduce SciKit-SurgeryGlenoid, an open source toolkit for the measurement of glenoid version. SciKit-SurgeryGlenoid contains implementations of the 4 most frequently used glenoid version measurement algorithms enabling easy and unbiased comparison of the different techniques. We present the results of using the software on 10 sets of pre-operative CT scans taken from patients who have subsequently undergone shoulder replacement surgery. We further compare these results with those obtained from a commercial implant planning software.

SciKit-SurgeryGlenoid currently requires manual segmentation of the relevant anatomical features for each method. Future work will look at automating the segmentation process to build an automatic and repeatable pipeline from CT or radiograph to quantitative glenoid version measurement.

## 1 Introduction

Correct understanding of the geometry of the glenoid (the socket of the shoulder joint) is key to successful planning of shoulder replacement surgery. This surgery typically involves placing an implant in the shoulder joint to restore joint function. The most relevant geometry is the glenoid version, which is the angular orientation of the glenoid surface relative to the long axis of the scapula in the horizontal (axial) plane. However, measuring the glenoid version is not straightforward and there are multiple measurement methods in the literature and used in commercial planning software.

Glenoid version can be computed from 2D radiographs or from 3D CT scans. The use of radiographs for glenoid measurement has been shown to be less reliable that CT based methods,^18^ so most modern approaches use CT scans for glenoid version measurement. Methods using CT scans can be divided into 2D methods that use a single axial slice to estimate glenoid version and 3D methods that use landmark points in multiple slices. 2D methods have been shown to be more susceptible to positional variance,^5^ however there is not as yet an agreed single method for glenoid version measurement. There are many papers comparing the use of different methods for measuring glenoid version or proposing new methods.^4, 7, 24, 25^ Different methods are also implemented by implant vendors and commercial software suppliers^1–3^ however, the exact methods used in each case are not published.

There is a need therefore for reliable open source implementations of the various methods for measuring glenoid version to enable further research comparing the methods. We have developed SciKit-SurgeryGlenoid to meet this need and herein present early results of SciKit-SurgeryGlenoid’s use on retrospectively gathered data.

## 2 Methods

### 2.1 Measuring Glenoid Version

SciKit-SurgeryGlenoid currently implements two 3D methods; the two-plane method described by Ganapathi et al.^13^ and the 3D corrected Friedman method described by Budge et al.^6^ SciKit-SurgeryGlenoid also implements two 2D methods; Friedman’s method^11^ and the vault method described by Matsumura et al.^16^ Each implementation can be accessed via a command line application which takes as input a file describing the anatomical position of the required landmark points.

**Figure 1:**
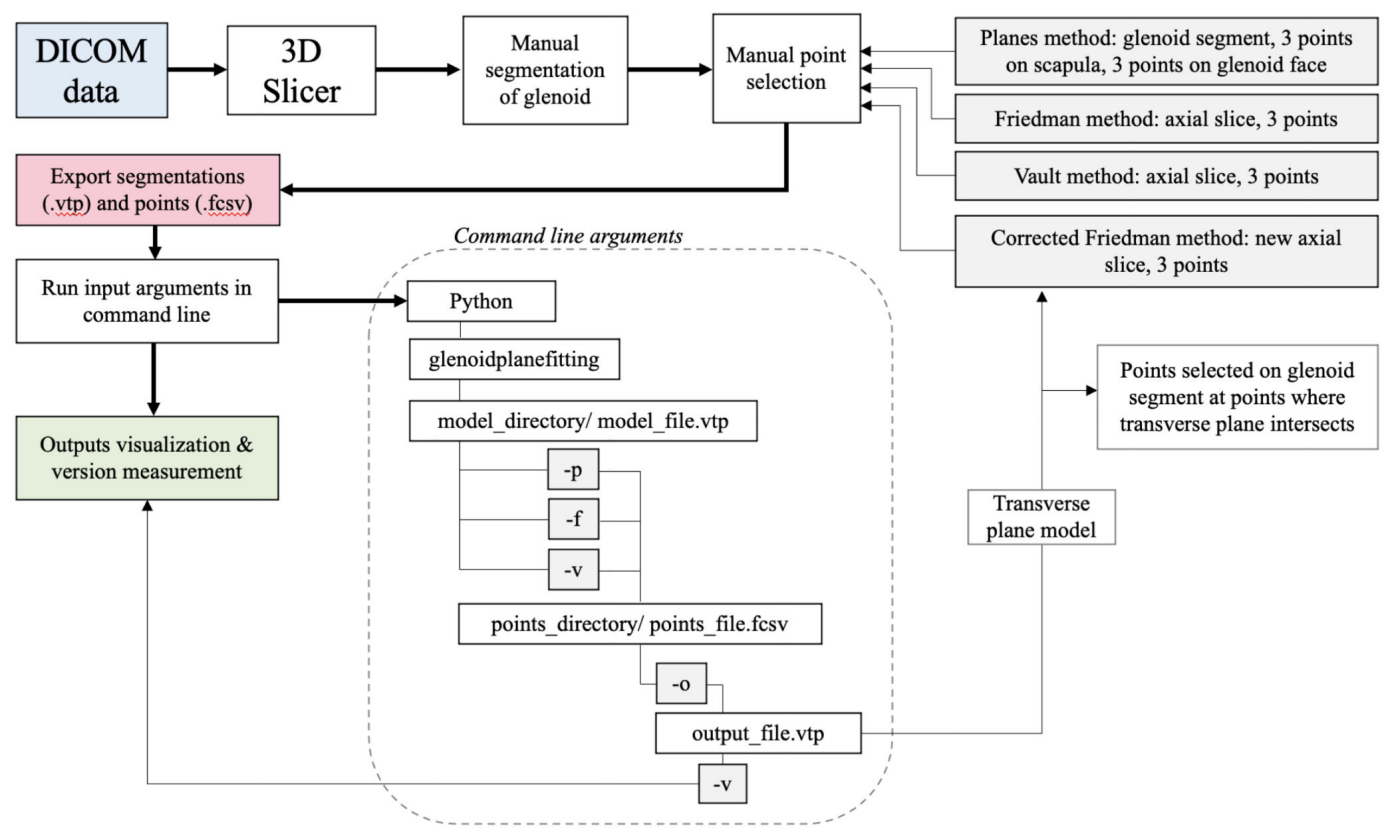
A workflow diagram describing the current workflow. Relevant landmarks are currently identified using 3DSlicer and passed to SciKit-SurgeryGlenoid via the command line. For the corrected Friedman method the points are manually identified a second time after identification of the new axial slice.

Although the longer term aim is to automate the identification of landmark points, SciKit-SurgeryGlenoid currently requires the landmark points to be manually identified. Figure 1 gives a graphical description of the work flow we used.

**Figure 2:**
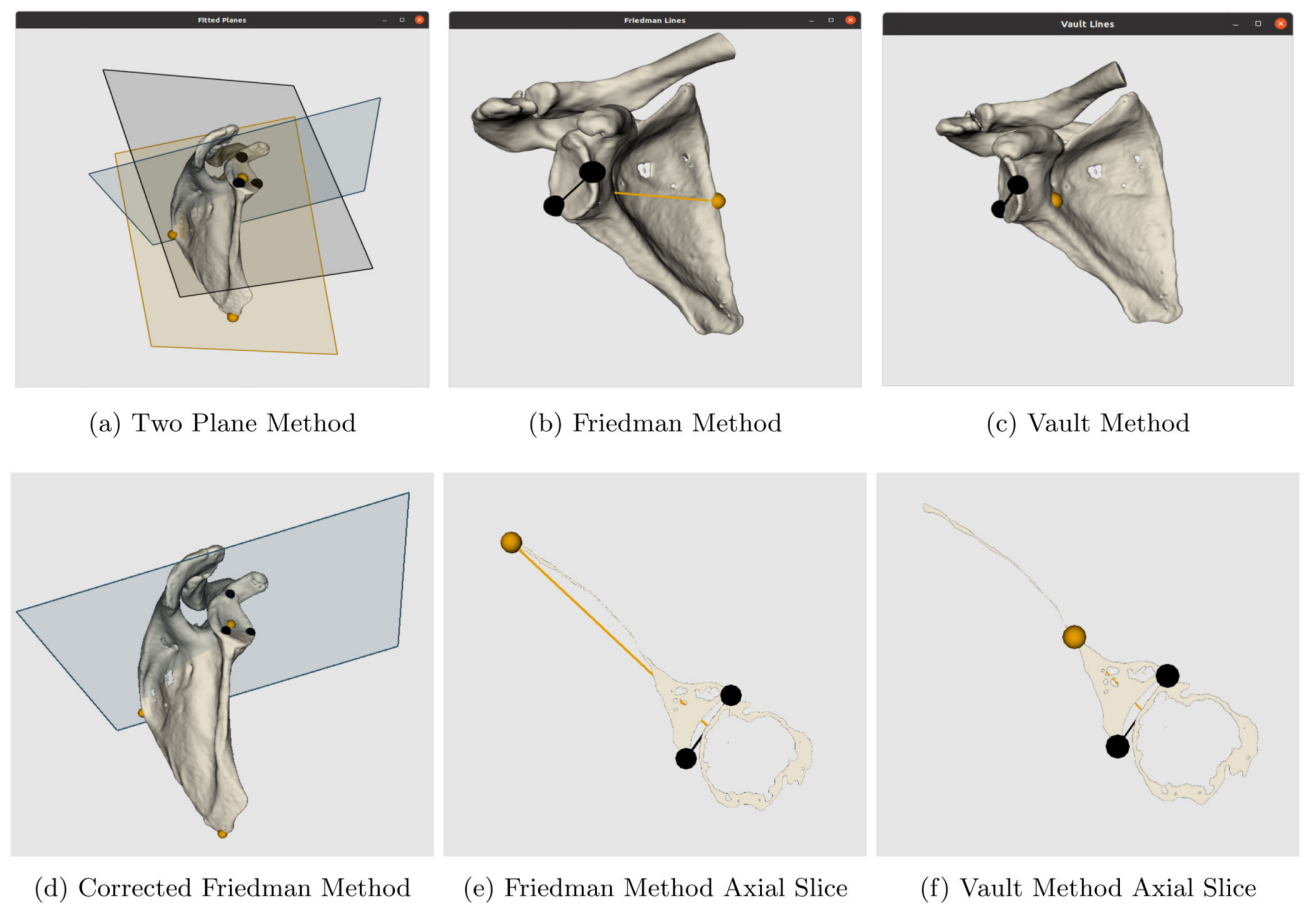
Visualisations of the 2 plane, Friedman and Vault methods and of new axial slice plane used for corrected Friedman method. Additional visualisations of the axial slices of the Friedman and vault methods to show point selection. By default, SciKit-SurgeryGlenoid uses the colour blind friendly palette defined by Wong^29^

The selection of the different landmark points for each method and calculations of version measurements were done as follows. For the two-plane method, 3 points were chosen for each plane. For the glenoid fossa plane the points selected were near the rim, one at the superior pole of the glenoid and two on the lower third of the glenoid anteriorly and posteriorly (black points on figure 2a). For the scapula plane, the 3 points included one at the center of the glenoid, another at the medial border of the scapula where the scapular spine intersects the scapular body, and a third at the inferior tip of the scapula (orange points on figure 2a). The glenoid version was then calculated as the angle between the plane of the glenoid fossa and the plane of the scapula.

For the Friedman and vault method 3 points were chosen to form 2 lines. Both require the same two points at the edges of the glenoid fossa anteriorly and posteriorly (black points on 2b and 2c). For the Friedman method the third point was selected at the tip of the scapula, while for the vault method it was at the tip of the scapular vault. The point selection was done on a 2D axial slice (see 2e and 2f). Therefore, slice choice is important and in this case was selected as the axial slice at which the coracoid process is no longer visible. The Friedman line was formed with the medial point on the scapula and the midpoint between the glenoid fossa points, while the vault line was formed with the tip of the scapular vault and the same midpoint. The second line was formed across the glenoid fossa in both methods. The version was then calculated as the angle between the two lines.

The corrected Friedman method requires the same anatomical landmark points as the conventional Friedman method, but on a corrected axial plane. This plane (blue plane in 2a) should be perpendicular to the scapular plane which is formed by the same 3 points as the scapular plane for the two-plane method (black plane in 2a). The new transverse scapular plane (blue plane in 2d) was used to generate a new 2D image slice on which the same conventional Friedman landmark points were selected.

### 2.2 Software Implementation with SciKit-Surgery

SciKit-SurgeryGlenoid is built on top of the SciKit-Surgery^27^ libraries which enabled rapid development and future deployment into a clinically useable application. Development was kick started using the SciKit-Surgery Python Template^9^ enabling the bulk of the development and testing to be performed during a 10 week summer internship with minimal prior experience of Python or software development. Use of the Python Template encourages good software development practice^22^ from the outset of the development process. SciKit-Surgery also includes template libraries for C++ projects.^10^

**Figure 3:**
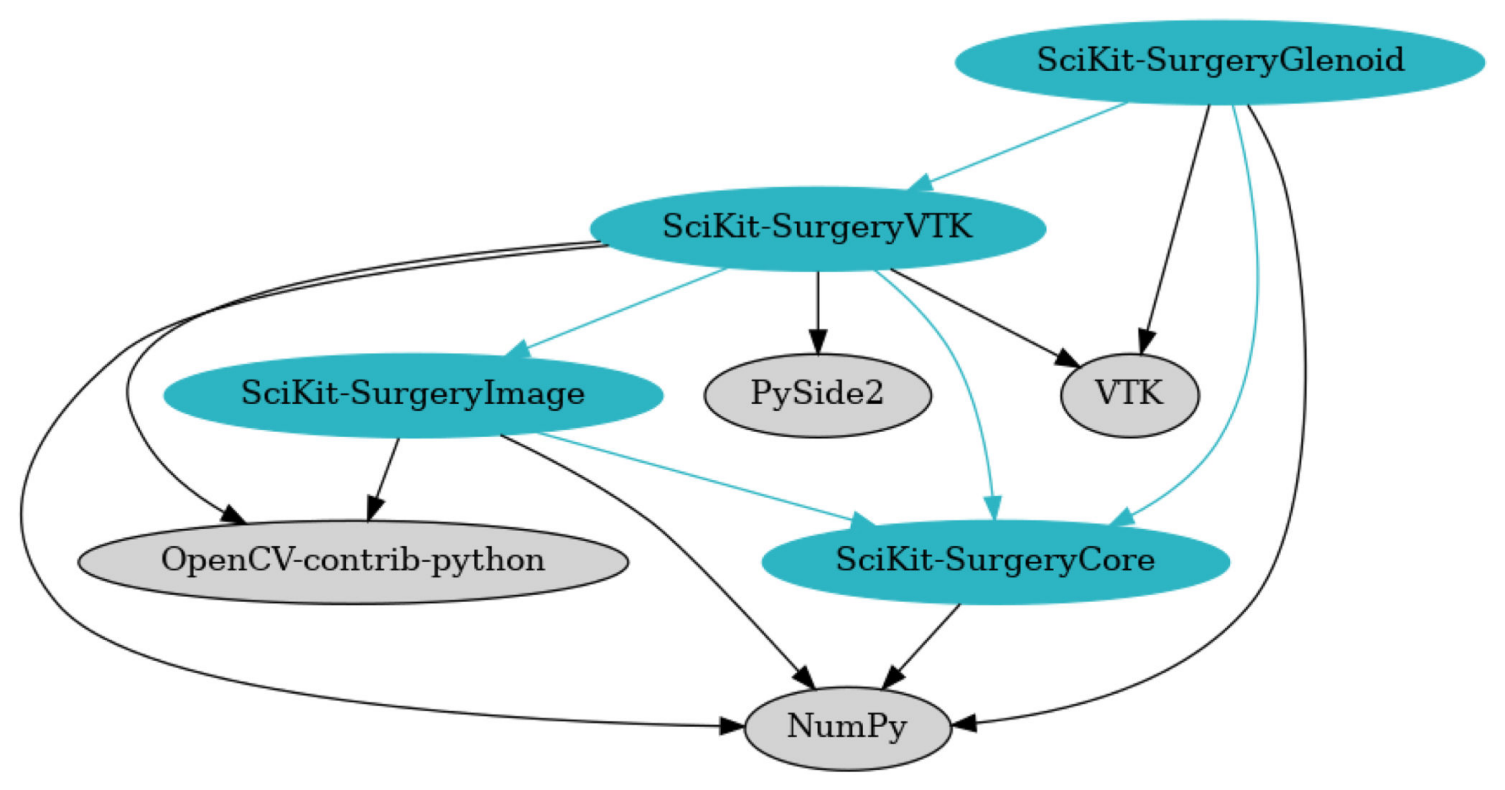
The dependency graph for SciKit-SurgeryGlenoid. SciKit-Surgery dependencies are shown in blue, whilst 3rd party dependencies are shown in grey.

SciKit-Surgery is made up of multiple Python libraries that can be assembled into varied applications for research in image guided surgery. Some current examples of SciKit-Surgery’s use include clinical guidance systems,^20^ research platforms for registration^26^ and ultrasound simulators.^28^ Figure 3 shows the immediate dependencies of SciKit-SurgeryGlenoid. The most significant dependencies are NumPy^14^ which is used for the version calculation, and VTK^21^ which is used for visualising the results. SciKit-SurgeryCore provides configuration helpers for the user interface. SciKit-SurgeryVTK provides some helpful loaders and shape primitives, but it may be useful to remove this dependency in the future as it would significantly simplify the dependency graph.

### 2.3 Experimental Validation

We tested the performance of SciKit-SurgeryGlenoid on 10 anonymised CT scans from patients eligible for shoulder replacement surgery. For each CT scan we performed segmentation and landmark annotation using 3DSlicer^15^ and processed the resulting segmentation using SciKit-SurgeryGlenoid.

Statistical analysis was performed using GraphPad Prism software version 8.0 for Mac *. The mean and standard deviation of each method was calculated and compared. Our current clinical practice uses planning software from DJO Surgical,^2^ so we compared the results from SciKit-SurgeryGlenoid with the results from the DJO Surgical software. Pearson’s correlation coefficient was determined between the commercial software and each method. A repeated measures ANOVA was performed to determine any significant differences in version measurements between the methods. Significance level for all analyses was set at 0.05.

## 3 Results

Table 1 presents the results of using SciKit-SurgeryGlenoid on 10 patients. The version measured using the planes method has a mean glenoid version of 8.9° (SD, 4.7°; range, 5° to 20.9°), while mean glenoid version for the 3D corrected Friedman method was 8.4°(SD, 6.5°; range, -4.0°to 16.9°). For the 2D methods, the mean glenoid version for the Friedman method was 9.4°(SD, 7.4°; range, -0.7° to 24°) and for the vault model was 12.3°(SD, 7.7°; range, 4° to 26°). In this case a positive value indicates retroversion while a negative value indicates anteversion of the glenoid. Overall, the 3D methods resulted in both lower mean version values as well as lower variability, while the 2D methods revealed a slightly higher variability.

**Figure 4:**
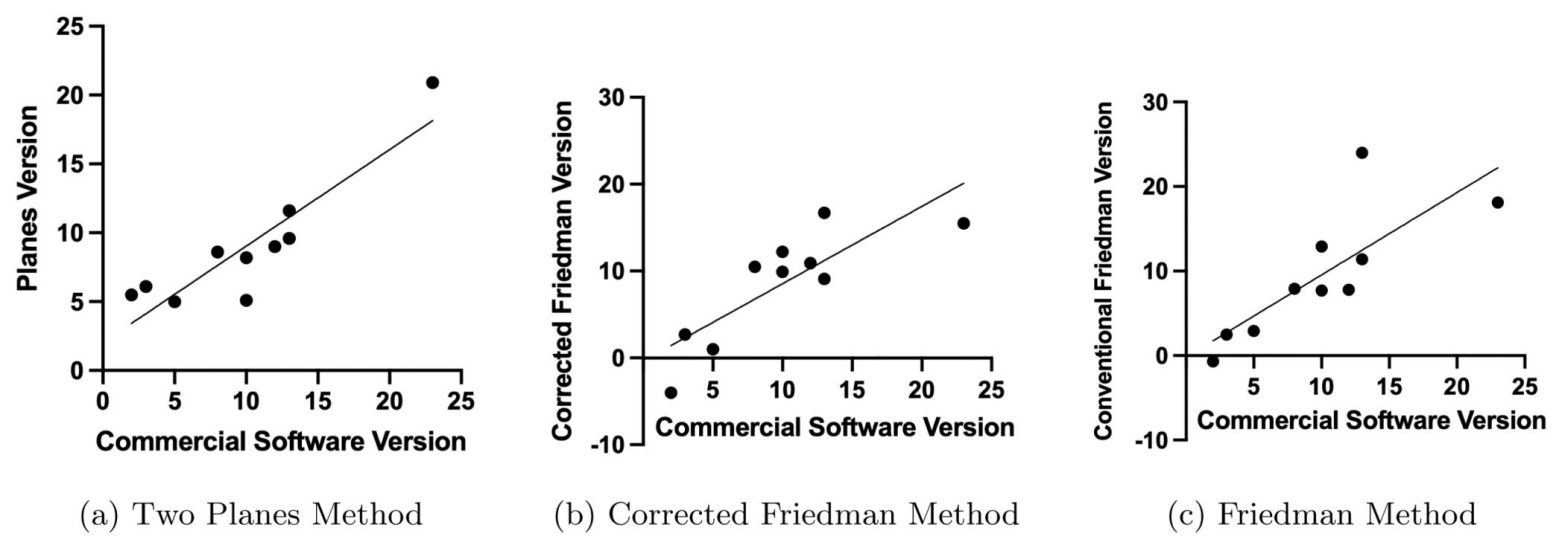
The Pearson correlation between the commercial software and the 3 methods that gave a statistically significant result.

The measurements using these methods were also compared with version measurements on the same 10 patients using a commercial software.^2^ The planes method (r = 0.90, p = 0.0004), corrected Friedman method (r = 0.83, p = 0.0034), and conventional Friedman method (r = 0.79, p = 0.0064) all showed significant correlation with the commercial software. The vault method did not show significant correlation (r = 0.59, p = 0.074). The mean difference between the methods were overall not significant (p > 0.05), except for the vault method (p = 0.03). Correlation plots are shown in Figure 4.

## 4 Discussion

There are several methods that have been proven to be accurate in preoperative measurement of the glenoid version. Specifically, 3D methods have become the standard as they provide a higher accuracy accounting for the positional errors during image acquisition (Budge et al.,^6^ Moineau et al.^17^). Testing the most common 2D and 3D methods using the SciKit-SurgeryGlenoid toolkit allowed for an evaluation of its effectiveness in comparing these methods. The early results presented are consistent with previously reported results (Matsumura et al.,^16^ Budge et al.,^6^ Ganapathi et al.^13^).

While the mean version did not show any significant difference between most methods, this could be due to the small sample size used in this case. However, it is notable that the 3D methods reveal slightly lower version means and lower standard deviations which could prove significant when more scans are analysed with additional observers.

From the Pearson correlation coefficient, significant correlation between the commercial software and 3 out of the 4 methods was seen. The vault method showing little correlation with the commercial software could be due to its much higher mean version value. The vault method tends to overestimate the glenoid version as has been previously reported by several studies (Cunningham et al.,^8^ Matsumura et al.^16^). The correlation tests prove however that there is good agreement between SciKit-SurgeryGlenoid and the commercial software already in use, indicating accuracy and credibility of this toolkit. While this can be a good indicator of reliability, further measurements using this software by different observers would be needed to be able to test inter and intra observer reliability. However, SciKit-SurgeryGlenoid proves to be promising in providing an unbiased way of comparing the many different methods available to measure glenoid version.

Limitations of this initial testing of SciKit-SurgeryGlenoid include the small sample size used. A study with a wider range of CT scans could reveal better understanding of the software’s reliability. Additionally, as points for each method were selected manually, there are some inaccuracies that arise which could be better understood with repeated measurements and multiple observers.

## 5 Conclusion

SciKit-SurgeryGlenoid provides a useful resource for shoulder arthroplasty. Future work could look at either automating the segmentation process using state of the art registration algorithms^12^ to create a fully automatic pipeline, or at integrating the library with 3DSlicer to create a “slicelet” based application, similar to our previous work^23^ in skull base navigation.

## Figures and Tables

**Table 1:** A comparison of the results of each method tested on 10 retrospective patients

Method	Mean Version	Standard Deviation
Two Planes	8.9°	4.7°
Corrected Friedman	8.4°	6.5°
Friedman	9.4°	7.4°
Vault	12.3°	7.7°
Commercial software	9.9°	6.1°

## Footnotes

* GraphPad Software, San Diego, CA, USA
